# Los aptámeros como novedosa herramienta diagnóstica y terapéutica y su potencial uso en parasitología

**DOI:** 10.7705/biomedica.4765

**Published:** 2020-08-20

**Authors:** Juan David Ospina

**Affiliations:** 1 Instituto Colombiano de Medicina Tropical, Universidad CES, Medellín, Colombia Universidad CES Instituto Colombiano de Medicina Tropical Universidad CES Medellín Colombia

**Keywords:** aptámeros de nucleótidos, aptámeros de péptidos, técnica SELEX de producción de aptámeros, anticuerpos monoclonales, parasitología, malaria, leishmaniasis, tripanosomiasis, amebiasis, aptamers, nucleotide, aptamers, peptide, SELEX aptamer technique, antibodies, monoclonal, parasitology, malaria, leishmaniasis, tripanosomiasis, amebiasis

## Abstract

Los aptámeros son secuencias de ADN o ARN de cadena sencilla que adoptan la forma de estructuras tridimensionales únicas, lo cual les permite reconocer un blanco específico con gran afinidad. Sus usos potenciales abarcan, entre otros, el diagnóstico de enfermedades, el desarrollo de nuevos agentes terapéuticos, la detección de riesgos alimentarios, la producción de biosensores, la detección de toxinas, el transporte de fármacos en el organismo y la señalización de nanopartículas.

El pegaptanib es el único aptámero aprobado para uso comercial por la *Food and Drug Administration* (FDA). Otros aptámeros para el tratamiento de enfermedades están en la fase clínica de desarrollo.

En parasitología, se destacan los estudios que se vienen realizando en *Leishmania* spp., con la obtención de aptámeros que reconocen la proteína de unión a poliA (LiPABP) y que pueden tener potencial utilidad en la investigación, el diagnóstico y el tratamiento de la leishmaniasis. En cuanto a la malaria, se han obtenido aptámeros que permiten identificar eritrocitos infectados e inhiben la formación de rosetas, y otros que prometen ser alternativas para el diagnóstico al detectar de forma específica la proteína lactato deshidrogenasa (PfLDH). Para *Cryptosporidium parvuum* se han seleccionado aptámeros que detectan ooquistes a partir de alimentos o aguas contaminadas. Para *Entamoeba histolytica* se han aislado dos aptámeros llamados C4 y C5, que inhiben la proliferación *in vitro* de los trofozoítos y tienen potencial terapéutico. Los aptámeros contra *Trypanosoma cruzi* inhiben la invasión de células LLC-MK2 (de riñón de mono) en un 50 a 70 % y aquellos contra *T. brucei* transportan moléculas tóxicas al lisosoma parasitario como una novedosa estrategia terapéutica.

Los datos recopilados en esta revisión destacan los aptámeros como una alternativa para la investigación, el diagnóstico y el tratamiento contra parásitos de interés nacional.

## Abstract (en)

Aptamers are single-stranded DNA or RNA sequences that adopt unique three-dimensional structures that allow them to recognize a specific target with high affinity. They can potentially be used for the diagnosis of diseases, as new therapeutic agents, for the detection of food risks, as biosensors, for the detection of toxins, and as drug carriers and nanoparticle markers, among other applications.

To date, an aptamer called pegaptanib is the only aptamer approved by the Food and Drug Administration (FDA) for commercial use. Other aptamers are in different clinical stages of development for the treatment of different diseases.

In parasitology, investigations carried out with parasites such as *Leishmania* spp. allowed the acquisition of aptamers that recognize the polyA-binding protein LiPABP and may have potential applications in research and diagnosis and even as therapeutic agents. Regarding malaria, aptamers have been obtained that allow the identification of infected erythrocytes or inhibit the formation of rosettes, along with those that provide promising alternatives for diagnosis by specifically detecting the protein lactate dehydrogenase (PfLDH). In *Cryptosporidium parvum* allow the detection of oocysts in contaminated food or water. In *Entamoeba histolytica*, two aptamers called C4 and C5, which inhibit the proliferation of trophozoites *in vitro* and have potential use as therapeutic agents, have been isolated.

Aptamers obtained against *Trypanosoma cruzi* inhibit the invasion of LLC-MK2 (from monkey kidney) cells by 50-70%, and in *T. brucei*, aptamers with the potential to transport toxic molecules to the parasitic lysosome were identified as a novel therapeutic strategy. The data collected in this review highlight aptamers as a novel alternative in the research, diagnosis, and treatment of parasites of national interest.

## Los aptámeros

Los aptámeros son secuencias de ADN o ARN de cadena sencilla, de 20 a 80 nucleótidos de longitud y con una región central variable que les permite adoptar formas estructurales tridimensionales únicas. Se ha establecido que pueden unirse a su blanco con una gran afinidad, con constantes de disociación del orden picomolar (pM) o nanomolar (nM) ^1^. Al igual que los anticuerpos monoclonales, los aptámeros tienen capacidad de reconocer moléculas antigénicas, por lo que también han sido llamados anticuerpos químicos ^2^. Ellington, *et al.*^3^*,* acuñaron por primera vez el nombre de *aptámero* a partir de la raíz latina *aptus,* es decir, “que encaja”, y la raíz griega *mers,* que significa “molécula”, por lo que el término aptámero quiere decir “molécula que encaja”.

En 1990, dos grupos diferentes de investigadores ^3^^,^^4^ demostraron la capacidad que tienen los ácidos nucleicos de formas estructurales tridimensionales únicas e intrincadas que les permiten reconocer con mucha sensibilidad y especificidad gran variedad de blancos inmunógenos y no inmunógenos, entre los que se destacan iones ^5^, toxinas ^6^, moléculas orgánicas pequeñas ^7^, antibióticos ^8^, aminoácidos ^9^, péptidos ^10^, carbohidratos ^11^, proteínas recombinantes ^12^, factores de crecimiento ^13^, virus ^14^, bacterias ^15^, parásitos ^16^, líneas celulares ^17^, hormonas ^18^, sustancias tóxicas ^19^ y compuestos activos de drogas ilegales ^20^, entre muchos otros.

**Figura 1:**
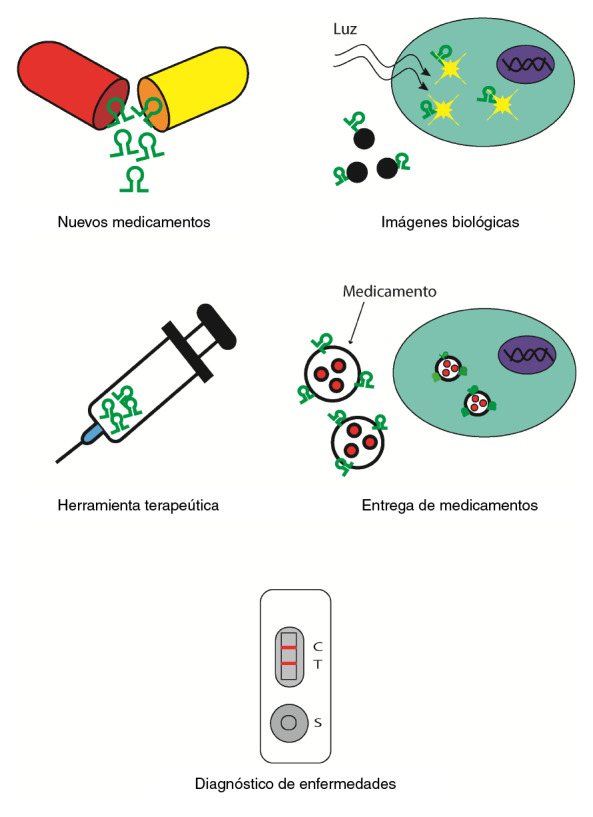
Los aptámeros se han usado, principalmente, para crear nuevos tipos de medicamentos, herramientas terapéuticas y plataformas para diagnosticar enfermedades, y en investigación, como detectores de moléculas en imágenes biológicas y como transportadores en la entrega de medicamentos.

Los usos de los aptámeros son tan variados como los blancos que pueden reconocer (figura 1). Los más estudiados, entre otros, han sido en el diagnóstico de enfermedades ^21^, como novedosa herramienta terapéutica ^22^, como transportadores de fármacos ^23^, en la marcación para imágenes biológicas ^24^, como biosensores ^25^, en la inspección de alimentos ^26^ y en la orientación de nanopartículas ^27^.

**Figura 2:**
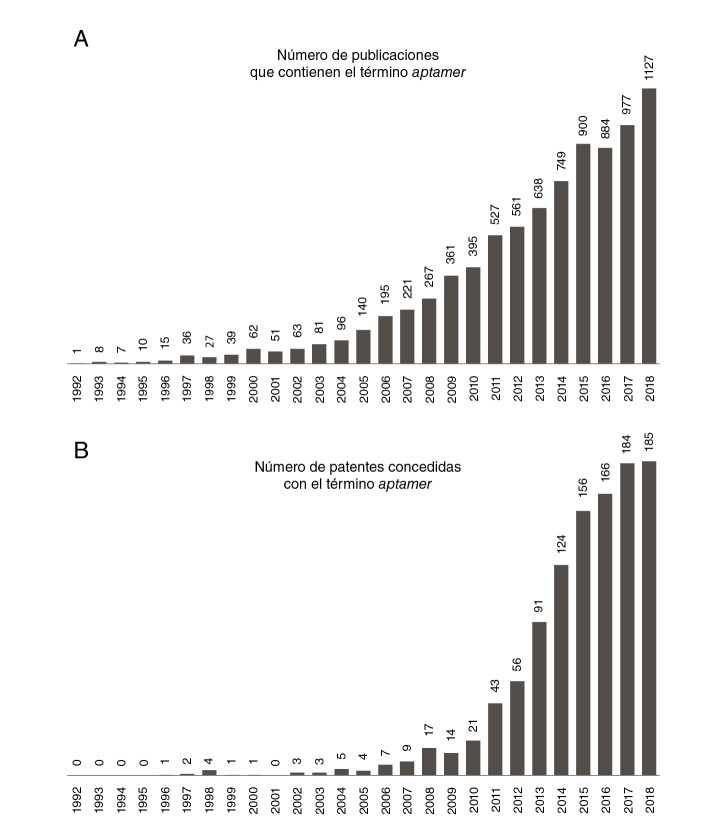
A) Línea de tiempo de publicaciones que contienen el término aptámero. Se realizó la búsqueda del término “aptamer” en PubMed. Los resultados se grafican como número de publicaciones por año desde 1992 hasta 2018. B) Patentes solicitadas que contienen el término aptámero. Los resultados se grafican como número de publicaciones por año desde 1992 hasta 2018.

Debido a que los aptámeros son relativamente nuevos y la técnica para obtenerlos, la *Systematic Evolution of Ligands by Exponential Enrichment* (SELEX) estuvo protegida por cuestiones complejas de propiedad intelectual ^28^^,^^29^, son pocos los artículos reportados hasta hoy en PubMed (https://www.ncbi.nlm.nih.gov/pubmed) que incluyen el término *aptamer* (~8.000 resultados), en comparación con otros temas que concitan mayor atención como *cancer* (~1’500.000 resultados) u *obesity* (~3’000.000 resultados). Una vez expiró la patente de la SELEX en el 2013, el número de publicaciones ha venido creciendo exponencialmente año tras año (figura 2).

Además, se buscaron las patentes concedidas con el término *aptamer* a nivel mundial en el sitio https://patents.google.com, usando como condiciones que el término se encontrara en el título y que las patentes hubieran sido publicadas entre 1992 y 2018. Se obtuvieron cerca de 1.000 resultados.

Curiosamente, el 81 % de las solicitudes provienen solo de tres países: 41 % de China, 21 % de Estados Unidos y 19 % de Corea del Sur. El resto proviene de la Unión Europea (9 %), Japón (9 %) y otros países (1 %).

En la figura 2 se señala el número de publicaciones que incluyen el término *aptamer* en el título, así como la cantidad de patentes que fueron concedidas entre 1992 y 2018. El comportamiento exponencial sugiere, en primer lugar, que tanto los investigadores como las empresas biotecnológicas y farmacéuticas han empezado a fijar su atención en estas novedosas moléculas y, por otro lado, que hay un interés particular en su uso comercial. Los aptámeros son relativamente fáciles de producir y, por su capacidad de interacción y reconocimiento de blancos complejos, pueden competir en el mercado con los anticuerpos monoclonales. En un estudio de *Markets and Markets* publicado en el 2015 (código BT3550), se sugiere que el mercado de los aptámeros alcanzará los USD$ 244,93 millones para el 2020 debido a su bajo costo y gran eficiencia, en comparación con los anticuerpos monoclonales ^30^.

Por otro lado, las infestaciones parasitarias continúan afectando principalmente a las personas más vulnerables que viven en áreas rurales tropicales con condiciones sanitarias y de salud deficientes, generalmente sin acceso a agua potable. Según la Organización Mundial de la Salud (OMS), solo de malaria -la parasitosis con más casos a nivel mundial- en el 2016 hubo 216 millones de casos, de los cuales 445.000 terminaron en muertes.

Los recursos para el diagnóstico, el tratamiento, el seguimiento, la prevención y la investigación de las infestaciones parasitarias son insuficientes, por lo que la mayoría se catalogan como enfermedades tropicales olvidadas o desatendidas. A ello se añade que, hasta el momento, no existe ninguna vacuna para su control y los medicamentos antiparasitarios producen graves efectos secundarios ^31^. Es urgente, entonces, buscar alternativas diagnósticas y terapéuticas de bajo costo, gran sensibilidad y especificidad, y fácil acceso.

En cuanto al uso de aptámeros en parasitología, se hizo una búsqueda en PubMed a nivel mundial con los términos [“aptamer” AND “parasite”]. Solo se encontraron 23 publicaciones, que se agruparon según el tipo de parásito y que serán enumeradas más adelante.

La escasa cantidad de publicaciones sobre los aptámeros en parasitología, refleja el desconocimiento de estas moléculas y de la técnica SELEX. Al parecer, falta una mayor divulgación para que los investigadores se interesen en los aptámeros, y se puedan desarrollar nuevas aplicaciones diagnósticas y terapéuticas en parasitología. La obtención de aptámeros permitiría mejorar enormemente el diagnóstico y el tratamiento de infestaciones parasitarias como una alternativa de bajo costo, y fácil producción y uso en regiones apartadas, ya que no requieren refrigeración, tienen larga duración y no se degradan tan fácilmente como los anticuerpos.

Con esta revisión se buscó dar a conocer los aptámeros, resaltar sus beneficios frente a otras moléculas con funciones similares, como los anticuerpos monoclonales, y explorar su potencial uso en el campo de la parasitología para mejorar el diagnóstico, la investigación y el desarrollo de nuevos medicamentos.

## Técnica SELEX

La técnica SELEX, en español evolución sistemática de ligandos por enriquecimiento exponencial, integra varias herramientas de biología molecular para la obtención de los aptámeros.

**Figura 3:**
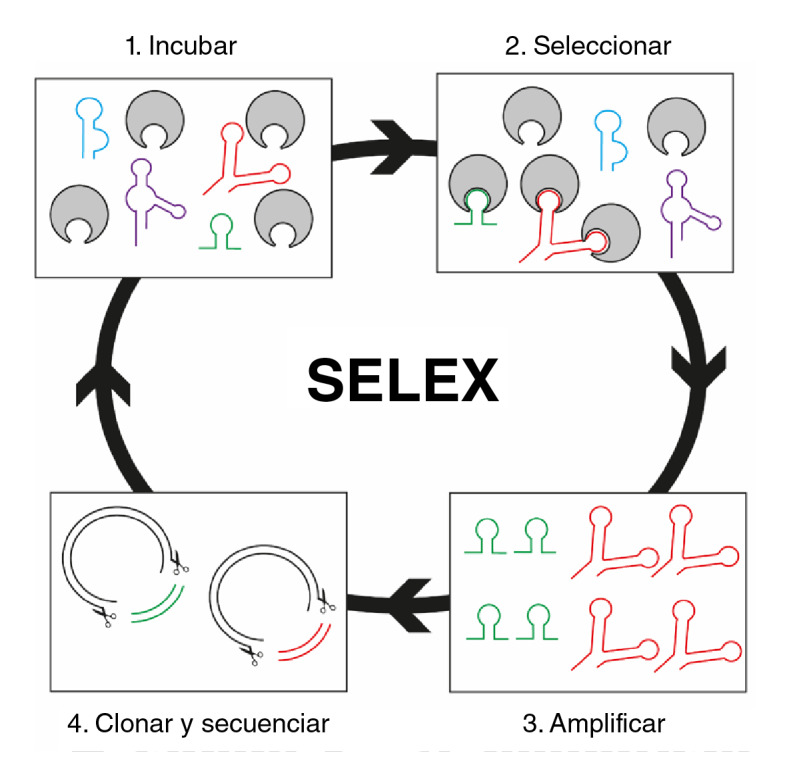
Técnica SELEX básica compuesta de cuatro pasos. 1) Incubación: la librería previamente diseñada es puesta en contacto con las moléculas blanco. 2) Selección: separación de los aptámeros que se unen a las moléculas blanco y descarte de aquellos que no lo hacen. 3) Amplificación: mediante PCR se obtienen múltiples copias de los aptámeros seleccionados. Se repiten los pasos 1, 2 y 3 hasta obtener aptámeros con gran afinidad y especificidad. 4) Clonación y secuenciación: una vez obtenidos los aptámeros afines, se clonan y se secuencian para conocer su composición de nucleótidos.

Para implementar la técnica, se necesita una librería de ADN o ARN que contenga una región variable en el centro de la secuencia (20-40 nucleótidos), y regiones conservadas en los extremos que permitan diseñar oligonucleótidos específicos para amplificar y seleccionar las secuencias que se unen al blanco. Los pasos básicos de la técnica SELEX son los siguientes (figura 3).

*Incubación.* Una vez obtenida la librería de oligonucleótidos (ADN o ARN), se pone en contacto con el blanco que se quiere reconocer, el cual puede estar anclado a una superficie o en suspensión.

*Selección.* Para los blancos que están anclados a una superficie, se hacen varios lavados, con el fin de descartar las secuencias no unidas. Si el blanco se encuentra en suspensión, es necesario centrifugar para separar las secuencias unidas de las no unidas. Una vez obtenidos los complejos de secuencia y blanco, este se debe separar para obtener únicamente las secuencias. Para esto existen varias estrategias, como el uso de detergentes, proteinasas y soluciones tampón, entre otras.

*Amplificación.* Posteriormente, mediante la reacción en cadena de la polimerasa (PCR) o PCR con transcripción inversa (RT-PCR), según la naturaleza del ácido nucleico, se amplifican las secuencias unidas para enriquecer la librería con aquellas que tienen capacidad de reconocer el blanco, las cuales se someten después a nuevas rondas de incubación y selección.

*Clonación y secuenciación.* Una vez se obtienen secuencias que alcancen la afinidad y especificidad deseadas, se procede a clonar y secuenciar para conocer su composición de nucleótidos.

Un paso adicional que mejora enormemente la especificidad de la técnica SELEX, es la “selección negativa*”.* Este paso consiste en poner las secuencias seleccionadas en contacto con blancos inespecíficos que no sean reconocidos. La afinidad de los aptámeros sometidos a selección negativa es hasta 10 veces mayor que la de los que no se someten a esta ^31^. Por ejemplo, incorporando la selección negativa a la técnica SELEX se han obtenido aptámeros que reconocen una línea celular cancerígena (glioblastoma U251, U87MG), pero no en el mismo tipo de células sanas ^32^. También, se han seleccionado aptámeros que reconocen positivamente células de un tipo de cáncer específico (cáncer de mama SK-BR3), pero no reconocen otras líneas celulares del mismo tipo de cáncer (MDA-MB-231 y MDA-MB-468) ^33^. La selección negativa permite, incluso, obtener aptámeros que reconocen proteínas con una mutación puntual ^34^ o isómeros de una misma proteína ^35^.

Como dato curioso, la técnica SELEX también se ha reconocido como una evolución *in vitro* debido a que la librería inicial de oligonucleótidos es filtrada hasta obtener los aptámeros más “aptos” o de mayor afinidad con un blanco específico. Como lo mencionaron Gold, *et al.*^4^, en su publicación de 1990 en *Science*: “SELEX puede ser solo el comienzo de la evolución en un tubo de ensayo”.

## Modificaciones de la técnica SELEX

Desde su creación en 1990, la técnica SELEX ha experimentado algunas modificaciones con el fin de optimizar el proceso. Por ejemplo, para reducir considerablemente el tiempo de obtención de los aptámeros, se implementó en el 2004 una modificación llamada SELEX por electroforesis capilar (CE- SELEX), la cual permite la separación más efectiva de los ácidos nucleicos unidos y no unidos, lo que reduce las rondas de selección a entre 1 y 4, en tanto que con la estrategia convencional se requieren de 15 a 20 rondas de selección ^36^.

Otra modificación pensada para mejorar la selección fue la implementación de la electroforesis capilar sin equilibrio de mezclas, Neceem (*NonEquilibrium Capillary Electrophoresis of Equilibrium Mixtures*), también llamada no-SELEX, que permite obtener aptámeros en cuestión de horas y no meses, como ocurre con el método convencional ^37^.

En el 2006, se desarrolló una técnica que acoplaba un sistema de microfluidos con la técnica SELEX (M-SELEX), lo cual reduce significativamente los costos y permite la automatización del proceso ^38^.

Al terminar los pasos de la metodología SELEX, es necesario analizar los ácidos nucleicos seleccionados. Tradicionalmente, se hacía mediante el método de secuenciación de Sanger, pero recientemente se ha optado por reemplazar el análisis clásico por una secuenciación de alto rendimiento (*High Throughput Sequencing*, HTS), lo que permite identificar las librerías después de cada ronda de selección, lo cual evidencia el enriquecimiento de ciertas secuencias, así como estandarizar y reducir el tiempo de selección. Este método ha sido llamado HTS-SELEX ^39^.

Muchas otras modificaciones se han introducido en la técnica SELEX para optimizar el proceso de obtención de aptámeros, las cuales pueden consultarse con mayor detalle en las revisiones publicadas por Kong, *et al.*^40^, y Zhuo, *et al.*^41^.

## Ventajas y desventajas de los aptámeros

**Figura 4:**
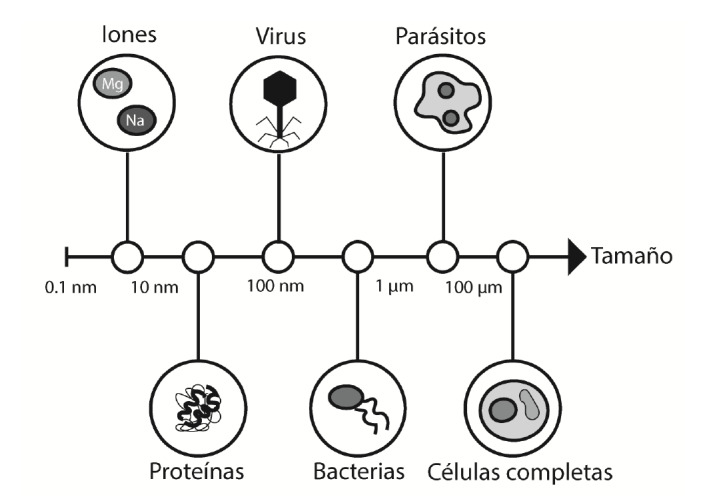
Variedad de blancos reconocidos por los aptámeros. La escala de tamaños muestra la capacidad de los aptámeros de reconocer moléculas que van desde 0,1 nm hasta otras más complejas, de 100 µm.

Al igual que los anticuerpos monoclonales, los aptámeros reconocen su blanco de forma específica y con gran afinidad. Una de sus ventajas frente a los anticuerpos monoclonales, es su capacidad de reconocer blancos de carácter no inmunógeno, lo que amplía enormemente sus potenciales usos (figura 4, cuadro 1). Otra ventaja reconocida de los aptámeros es que, al ser obtenidos *in vitro,* requieren de una menor manipulación y, por lo tanto, la producción conlleva menos riesgo de contaminación, menor tiempo de producción y una mejor reproducibilidad, con lo que aumenta enormemente la posibilidad de escalar el proceso de producción.

**Cuadro 1:** Ventajas y desventajas de los aptámeros

Aptámeros	
Ventajas	Desventajas
• No requieren modelos biológicos.	• Su desarrollo apenas comienza.
• Tiempo de producción breve (1 a 3 meses)	• Farmacocinética variable
• Fácil de escalar	• Proclives a filtración renal
• Pueden modificarse químicamente para evitar degradación.	• Corta vida media
• Largo tiempo de almacenamiento a temperatura ambiente	
• Se unen a una amplia variedad de blancos inmunógenos y no inmunógenos.	• Los aptámeros no modificados químicamente se degradan fácilmente en el suero.
• Menor tamaño para acceder a tejidos o células (12-15 kDa)	
• Bajo costo de producción	• La técnica SELEX está protegida bajo condiciones de propiedad intelectual.
• La síntesis química disminuye la variabilidad entre lotes.	
•La secuencia de nucleótidos se almacena para posteriores producciones.	
• Especificidad y afinidad iguales o mejores que las de los anticuerpos monoclonales	
• La patente de la técnica SELEX expiró en el 2013.	

Los datos de secuenciación que se obtienen con la técnica SELEX pueden almacenarse en una computadora y estar siempre disponibles hasta el momento en que se requiera su síntesis química, lo que representa una gran ventaja en cuanto a ahorro de almacenamiento. La síntesis química también representa una disminución en la variabilidad entre lotes. Una vez sintetizados, los aptámeros pueden almacenarse a temperatura ambiente (liofilizados) y, cuando sean reconstituidos, pueden desnaturalizarse y renaturalizarse sin perder su capacidad de reconocimiento. Químicamente, también pueden modificarse para hacerlos resistentes a la actividad de las nucleasas y alargar así su vida media ^42^. Su tamaño es, aproximadamente, de 12 a 15 kDa, lo que permite su penetración en tejidos o células de difícil acceso, en tanto que los anticuerpos monoclonales tienen un tamaño aproximado de 150 a 170 kDa que no les permite penetrar en ciertos tejidos.

Las principales desventajas de los aptámeros son su degradación por la acción de las nucleasas, la filtración renal y su corta vida media. Sin embargo, hoy en día pueden hacerse modificaciones químicas que mejoran enormemente su rendimiento, por ejemplo, acoplando polietilenglicol (PEG) al extremo 5’ o a biotina en el extremo 3,’ lo que aumenta su peso molecular y evita su filtración ^43^. Para evitar la degradación por la acción de las nucleasas, es posible hacer modificaciones en el anillo de azúcar, usar nucleótidos invertidos y modificaciones en la unión azúcar-fosfato que pueden incluirse en la estructura química ^42^.

Otras modificaciones, como la inversión del 3,’ 2’-fluoro, 2’-O-metil y la sustitución del 2’ mediante aminoácidos captados por ácido nucleico bloqueado, mejoran la estabilidad metabólica, la afinidad de la interacción y la filtración renal ^44^^,^^45^.

## ¿Cómo reconocen los aptámeros su blanco?

Se ha determinado la estructura cristalográfica de múltiples complejos aptámero-blanco ^46^^-^^48^. Dichos complejos revelan la importancia de los diferentes *plegamientos* que pueden adoptar los aptámeros: tallos-bucles, horquillas, protuberancias, pseudonudos y G-cuádruplex, que les permite reconocer su blanco por medio de enlaces de hidrógeno, interacciones hidrofóbicas, interacciones de van der Waals y apilamiento aromático, entre otros ^49^. En la estructura cristalográfica de los aptámeros de ARN unidos a su blanco, resalta la capacidad que tienen de adoptar conformaciones intrincadas que les permiten reconocer grandes superficies y unirse con gran afinidad a blancos que naturalmente no se unirían al ARN ^50^.

Lo que queda claro del análisis de los complejos aptámero-blanco es que, por lo general, los aptámeros no dependen directamente de su secuencia primaria para la interacción con su blanco sino de su estructura tridimensional. El reconocimiento y la unión al blanco implican interacciones tridimensionales dependientes de la forma, así como interacciones hidrófobas, apilamiento de bases e intercalación. Se requieren diversos cambios de conformación tanto del aptámero como del blanco para producir un “ajuste inducido”, el cual es difícil de predecir, pero esencial para que se dé la interacción con gran afinidad ^51^^-^^53^. Precisamente, los aptámeros no “reconocen” su blanco; el término correcto sería que “encajan” con su blanco, de allí su definición de molécula que encaja.

## Aptámeros naturales (ribointerruptores)

En el 2002, casi una década después del descubrimiento de los aptámeros en 1990, se encontró que existían en las células moléculas de ARN con funciones reguladoras naturales, las cuales recibieron el nombre de ribointerruptores (*riboswitches*) o aptámeros naturales, ya que reconocen con gran especificidad su blanco con base en el cambio de su estructura tridimensional. La estructura de los ribointerruptores se divide en dos partes: un aptámero que se une directamente al metabolito y una plataforma de expresión que es la encargada de regular la expresión génica uniéndose directamente a los ARN mensajeros en la región no codificadora 5’ UTR (5’ *UnTranslated Region*).

Los primeros ribointerruptores se identificaron en bacterias actuando como sensores intracelulares de derivados de vitaminas. Lo que hacen los ribointerruptores es censar estos derivados y modular la transcripción de los ARN mensajeros induciendo alteraciones en la estructura secundaria, lo que afecta la expresión de las proteínas que están involucradas en la biosíntesis, el transporte y la utilización de los metabolitos ^54^.

Con el pasar de los años, se ha reconocido que los ribointerruptores participan en el control de la expresión génica en numerosas especies de bacterias, arqueas, plantas, hongos y algas ^55^^,^^56^. Se ha descubierto también que, más allá de regular directamente la transcripción de los ARN mensajeros, también pueden regular su traducción, empalme y estabilidad ^57^.

## Usos actuales de los aptámeros

El primer aptámero aprobado para su uso comercial por la *Food and Drug Administration* (FDA) fue el pegaptanib. En el 2004 fue declarado eficaz y se conoce comercialmente como Macugen™ (Eyetech Pharmaceuticals, Pfizer, New York, NY, USA). Este aptámero se usa para el tratamiento de la degeneración macular asociada con la edad. Se administra en inyecciones intravítreas cada seis semanas durante 48 semanas. El pegaptanib actúa como antagonista del factor de crecimiento endotelial vascular (*Vascular Endothelial Growth Factor*, VEGF), que es el causante de la angiogénesis exacerbada en esta enfermedad ^58^^,^^59^.

Para hallar otros aptámeros que están siendo ensayados y sus potenciales usos, se hizo la búsqueda de estudios clínicos en el sitio https://clinicaltrials. gov/ct2/home. Los criterios de búsqueda fueron que se estuvieran llevando a cabo en cualquier parte del mundo con la aprobación de la FDA y contuvieran en el título el término *aptamer.* Se encontraron 36 estudios, de los cuales uno se encontraba en fase IV (pegaptanib, ya aprobado), siete en fase III, 13 en fase II, 14 en fase I, y uno en fase I temprana.

En el cuadro 2 se pueden consultar los aptámeros más representativos, la enfermedad que buscan tratar y la fase clínica en la que se encuentran. La lista completa se puede consultar en el archivo suplementario 1.

**Cuadro 2:** Aptámeros en fases de desarrollo clínico

Aptámero	Blanco	Enfermedad	Fase clínica
Pegaptanib™ (Pfizer)	Factor de crecimiento vascular endotelial (VSGF)	Degeneración macular relacionada con la edad	Aprobado por la FDA Fase IV
REG1™ (Regado Biosciences)	Factor de coagulación IXa	Enfermedad de la arteria coronaria	Fase III
E10030™ (Ophthotech Corporation)	Factor de crecimiento derivado de plaquetas (PDGF)	Degeneración macular relacionada con la edad	Fase III
AS1411™ (antisoma)	Nucleolina	Leucemia mieloide aguda	Fase II
REG1™ (Regado)	Factor de coagulación IXa	Intervención coronaria percutánea	Fase II
ARC1779™ (Archemix)	Dominio A1, factor de von Willebrand	Microangiopatías trombóticas y enfermedad de la arteria carótida	Fase II
NOX-E36™ (Noxxon Pharma)	Citocina CCL2	Diabetes mellitus de tipo 2	Fase II
NU172™ (ARCA)	Trombina	Derivación cardiopulmonar para mantener el estado estable de la anticoagulación	Fase II
ARC19499™ (Baxter)	Inhibidor de la vía del factor tisular (TFPI)	Hemofilia	Fase I
ARC1905™ (Ophthotech)	Componente 5 del complemento	Degeneración macular relacionada con la edad	Fase I

## Aptámeros en parasitología

Es evidente la necesidad de nuevos agentes terapéuticos para el tratamiento de las infestaciones parasitarias que afectan al ser humano. A pesar de los avances en la secuenciación genómica de muchos parásitos, de los estudios de transcriptómica y proteómica, y del fortalecimiento de las demás ómicas que han derivado en la identificación de nuevos blancos terapéuticos, hasta hoy no se cuenta con nuevos medicamentos ni ninguna vacuna, lo cual se debe a la gran cantidad de tiempo requerido para el cribado de compuestos químicos contra los potenciales blancos identificados.

El actual método de elección para disminuir el tiempo y el costo del cribado *in vitro* es el análisis *in silico* (*virtual screening*), lo que permite filtrar los mejores compuestos para, posteriormente, ser probados *in vitro*. Los candidatos más prometedores se someten a modificaciones químicas para mejorar parámetros como la toxicidad y la estabilidad, ser probados en modelos animales y, por último, ser sometidos a estudios clínicos.

La técnica SELEX se presenta como una novedosa alternativa para el cribado de múltiples moléculas (10^12^-10^14^ ADN o ARN) en corto tiempo, con el fin de obtener aptámeros de gran afinidad y especificidad contra un blanco específico.

Actualmente, la técnica SELEX se usa en la obtención de aptámeros específicos contra diferentes blancos en parásitos como *Plasmodium falciparum*, *Entamoeba histolytica*, *Leishmania infantum*, *Cryptosporidium parvum*, *Trypanosoma cruzi* y *T. brucei.*

## Aptámeros contra *Plasmodium* spp.

*Plasmodium* spp. es el agente causal de la malaria y el parásito que más casos y muertes causa alrededor del mundo. En la fase eritrocitaria de su ciclo de vida, el parásito infecta los glóbulos rojos del huésped, los cuales expresan la proteína de membrana de eritrocitos 1 (PfEMP1) que, una vez en la membrana del eritrocito, se une a múltiples receptores celulares humanos como el heparán sulfato (HS), la ICAM-1, la CD36 y la CsA ^60^. Los eritrocitos infectados se adhieren al endotelio vascular y forman rosetas con eritrocitos no infectados; posteriormente, se produce la aglutinación mediada por plaquetas, lo que puede ocasionar complicaciones como la malaria cerebral.

La PfEMP1 le permite al parásito evadir la reacción inmunitaria debido a la variación antigénica, ya que se compone de un dominio llamado ligando de unión a Duffy (*Duffy Binding Ligand*, DBL) y regiones interdominio ricas en cisteína. La PfEMP1 es codificada por 60 genes *var* que son intercambiados regularmente por el parásito, generando variación antigénica en la superficie de los glóbulos rojos infectados. Cada uno de los 60 genes *var* expresa una PfEMP1 diferente.

Barfod, *et al.*^61^, utilizaron la técnica SELEX para obtener aptámeros de ARN específicos con modificaciones en 2’-fluoro desoxiadenosina que tienen una vida media de 15 horas en suero contra el N-terminal de uno de los dominios DBL (DBL1α), el cual es conservado en casi todas las diferentes proteínas PfEMP1 ^62^. Los aptámeros de ARN obtenidos tras ocho rondas de selección tienen la capacidad de reconocer la proteína recombinante DBL1α de forma específica y, por lo tanto, los eritrocitos infectados con *P. falciparum.* Asimismo, tienen la capacidad de interrumpir la formación de rosetas (35 % de reducción) *in vitro* del clon FCR3S1.2, de reconocida capacidad formadora de rosetas, en eritrocitos infectados y no infectados.

Por otro lado, Birch, *et al.*^63^, seleccionaron aptámeros de ARN con una novedosa modificación de la técnica SELEX mediante el acoplamiento a una plataforma inercial de microfluidos, la I-SELEX (*Inertial microfluidic SELEX*). Se seleccionaron aptámeros contra los glóbulos rojos infectados con *P. falciparum*. Tras cinco rondas de selección, obtuvieron un set de aptámeros que reconocen diferentes epítopos presentes únicamente en la superficie de los glóbulos rojos infectados. Estos aptámeros podrían ser útiles para el desarrollo de nuevas alternativas diagnósticas, tratamientos complementarios y vacunas, que ayuden al control de la infección.

Uno de los marcadores diagnósticos más importantes de *Plasmodium* spp. es la lactato deshidrogenasa (pfLDH). Cheung, *et al.*^64^^,^^65^, obtuvieron aptámeros de ADN acoplados a nanopartículas de oro que reconocen diferencialmente la pLDH de *P. falciparum*, con una constante de disociación en un rango de 42 a 59 nM, pero no así la pLDH de *P. vivax* y la LDH humana. Los aptámeros obtenidos pueden detectar el parásito en muestras de pacientes con paludismo y prometen ser una alternativa para el diagnóstico específico de especie, lo cual derivó en la creación de una prueba portátil de microfluidos de captura de enzimas por medio de aptámeros, la APTEC (*Aptamer-Tethered Enzyme Capture*) (APTEC) ^66^, que podría complementar o reemplazar la prueba que se usa regularmente con anticuerpos (inmunoensayo de detección de HRP2).

También, se han probado aptámeros de ADN como agentes terapéuticos para bloquear vías metabólicas de *Plasmodium* spp. y de desintoxicación mediante el grupo heme, que es el blanco de compuestos como la cloroquina que, al ser inhibida, induce la toxicidad del parásito. Niles, *et al.*^67^, obtuvieron aptámeros de ADN que inhiben de forma eficiente la vía de desintoxicación del grupo heme, que deriva en la toxicidad y la inhibición del crecimiento de los parásitos *in vitro*.

## Aptámeros contra *Entamoeba histolytica*

*Entamoeba histolytica* es el agente causal de la amebiasis humana y es el tercer parásito responsable de muertes en el mundo después de *Plasmodium* spp. y *Schistosoma* sp*.* Según la OMS, se estima que el 10 % de la población se encuentra infectada con este parásito, que causa cerca de 100.000 muertes al año.

La regulación de la expresión génica es un paso fundamental para todos los organismos eucariotas. En este proceso, la poliadenilación del extremo 3’ UTR de los ARN mensajeros tiene un papel fundamental ya que, si no son poliadenilados, los transcritos no pueden ser exportados al citoplasma y, por lo tanto, no se lleva a cabo su traducción.

Para que la poliadenilación ocurra, son necesarios múltiples complejos proteicos que reconocen secuencias propias de los ARN mensajeros. Varios de estos complejos son fundamentales para que este proceso se lleve a cabo de forma eficiente. Los más importantes son el factor específico de corte y poliadenilación (CPSF), el factor estimulante del corte (CstF), el factor de corte Im (CFIm), el factor de corte IIm (CFIIm), la poliadenilato polimerasa (PAP) y la proteína de unión a poliadelinato (PABP). El CFIm se compone de varias subunidades (72, 68, 59, y 25 kDa), de las cuales la de 25 kDa ha sido reconocida como fundamental para la selección de sitios de poliA, el reclutamiento de la maquinaria de poliadenilación, el corte y la poliadenilación ^68^. Se ha establecido que la proteína homóloga EhCFIm25 en *E. histolytica* es fundamental para la supervivencia del parásito ya que, al ser inhibida, se acelera la proliferación y, por lo tanto, la muerte celular ^69^^).^

En el 2018, se reportó por primera vez la selección de dos aptámeros de ARN tras siete rondas de SELEX, los cuales se unen de forma específica a la proteína EhCFIm25, sea esta recombinante o endógena. Los aptámeros evitan que la EhCFIm25 lleve a cabo su función en el ARN como reguladora del corte y la poliadenilación, lo que deriva en la muerte del parásito *in vitro,* por lo que representa una alternativa como herramienta terapéutica para el control de la amibiasis humana ^12^.

## Aptámeros contra *Leishmania* spp.

La leishmaniasis es una enfermedad tropical desatendida. Está presente en 102 países en cuatro continentes y ocasiona diferentes síntomas según la especie infecciosa. Sus manifestaciones incluyen desde úlceras en la piel (*Leishmania major*, *L. tropica*, *L. braziliensis* y *L. mexicana*) hasta lesiones mucosas (*L. braziliensis*) y viscerales (*L. donovani, L. chagasi* y *L. infantum*).

La proteína histona H2A se considera una excelente candidata para el diagnóstico de la leishmaniasis. Las histonas están muy conservadas en su región globular, pero presentan una gran divergencia en los dominios N y C terminales, los cuales se han estudiados con fines diagnósticos y terapéuticos. Ramos, *et al.*^70^, tras tres rondas de selección mediante la técnica SELEX, obtuvieron una población de aptámeros de ADN a la cual denominaron SELH2A. Esta reconoce de forma específica el antígeno H2A de *L. infantum* con un bajo límite de detección (50 ng). Posteriormente, estandarizaron una prueba de oligonucleótidos ligados a enzimas (ELONA, *Enzyme-linked oligonucleotide assay*), una *Western blot* y una *slot blot*, para detectar específicamente dicha histona H2A. Estas técnicas podrían usarse comercialmente para fortalecer el diagnóstico de esta parasitosis, especialmente de su forma visceral.

Posteriormente, Martín, *et al*. ^71^, aislaron dos aptámeros, el AptLiH2A#1 y el AptLiH2A#2, a partir del grupo (*pool*) SELH2A previamente obtenido, los cuales reconocen H2A con una constante de disociación en un rango muy bajo, del orden nanomolar (nM). Estos aptámeros purificados pueden sintetizarse, por lo que serían más prácticos a la hora de desarrollar una nueva herramienta diagnóstica.

Por otro lado, también se han identificado aptámeros de ADN contra la proteína PABP de *L. infantum* que, como ya se mencionó*,* es una proteína fundamental para el proceso de corte y poliadenilación de los 3’UTR de los ARN mensajeros. Luego de cuatro rondas de selección, se purificaron tres aptámeros: ApPABP#3, ApPABP#7 y ApPABP#11, los cuales reconocen la LiPABP con gran afinidad (50 nM). Además, el ApPABP#11 interrumpe la unión de la LiPABP a la cola de poliA de los ARN mensajeros *in vitro*. Estos aptámeros podrían usarse en investigación para purificar la LiPABP y para diagnóstico; incluso, el ApPABP#11 podría usarse como agente terapéutico debido a su capacidad de inhibir la unión de la LiPABP a la cola de poliA ^72^.

## Aptámeros contra *Cryptosporidium parvum*

La infestación por *Cryptosporodium* spp. se considera un problema de salud pública emergente, pues causa cuadros de gastroenteritis en personas inmunocompetentes, los cuales resultan más graves cuando hay compromiso del sistema inmunitario, especialmente por el HIV.

Iqbal, *et al.*^73^, aislaron 14 aptámeros de ADN después de diez rondas de selección, los cuales reconocen con gran afinidad ooquistes de *C. parvum* con una constante de disociación en un rango bajo de nM y tienen un gran potencial para detectar agua o alimentos contaminados, lo cual fortalecería los métodos diagnósticos tradicionales (microscopía y PCR) que actualmente se usan.

## Aptámeros contra *Trypanosoma* spp.

La tripanosomiasis es una enfermedad provocada por parásitos protozoarios hemoflagelados del género *Trypanosoma*. Las dos especies de mayor relevancia para el hombre causan dos enfermedades diferentes: *T. cruzi* ocasiona la tripanosomiasis americana o enfermedad de Chagas, la cual se transmite al ser humano principalmente por las heces u orina de insectos triatominos, y *T. brucei* causa la tripanosomiasis africana o enfermedad del sueño, la cual se transmite al ser humano por la picadura de la mosca tse-tse.

## Aptámeros contra *Trypanosoma cruzi*

El primer reporte de la utilización de la técnica SELEX contra *T. cruzi* permitió aislar aptámeros de ARN tras ocho rondas de selección, los cuales fueron dirigidos contra los receptores parasitarios en la matriz extracelular: laminina, fibronectina, trombospondina y heparán sulfato, para bloquear la interacción entre parásito y huésped ^74^. Se obtuvieron constantes de afinidad que oscilaron entre 40 y 400 nM, y se inhibió en un 50 a 70 % la invasión de células LLC-MK_2_ (de riñón de mono) en una concentración de 1µM. Los autores aclararon que no se inhibió al 100 % la invasión celular *in vitro* probablemente porque otros receptores no identificados podrían estar involucrados en este proceso.

Por otro lado, Nagarkatti, *et al.*^75^, obtuvieron aptámeros de ARN estables en suero que se unen a tripomastigotes vivos de *T. cruzi* con una afinidad en un rango de 8 a 25 nM. Uno de los aptámeros obtenidos (Apt68) reconoce con gran especificidad los tripomastigotes de *T. cruzi,* aunque no así otros tripomastigotes de parásitos relacionados, como *L. donovani* y *T. brucei*. El Apt68 fue inmovilizado en fase sólida y se demostró que puede capturar tripomastigotes con una concentración menor de cinco parásitos en 15 ml de sangre total, los cuales se pueden observar bajo el microscopio óptico en forma de agrupaciones que se pueden amplificar posteriormente por PCR en tiempo real para confirmar su identidad.

En este mismo estudio y tras 10 rondas de SELEX, se obtuvieron aptámeros de ARN contra antígenos excretados (*Trypomastigote Excreted- Secreted Antigens,* TESA) que son liberados en la sangre del huésped infectado y se han estudiado como potenciales biomarcadores. Los aptámeros obtenidos fueron marcados con biotinilo y reconocieron los TESA en plasma de ratones infectados con *T. cruzi* mediante una prueba de aptámeros ligada a enzimas (*Enzyme-Linked Aptamer Sorbent Assay*, ELASA). Uno de los aptámeros aislados, el Apt-L44, pudo detectar estos biomarcadores circulantes tanto en la fase aguda, 7 a 28 días después de la infección, como en la fase crónica, después de 55 a 230 días, por lo que se consolida como una potencial herramienta para la detección de *T. cruzi* en sangre de mamíferos infectados.

## Aptámeros contra *Trypanosoma brucei*

En *T. brucei* se han utilizado aptámeros de ARN como potenciales moléculas transportadoras introducidas por endocitosis para después unirse a una determinada proteína blanco localizada dentro del bolsillo flagelar.

Específicamente, el aptámero de ARN llamado 2-16 es capaz de reconocer el lisosoma del parásito. Este aptámero podría usarse como transportador de moléculas tóxicas hasta el lisosoma parasitario, como novedosa estrategia terapéutica ^76^.

## Conclusiones y perspectivas

Los aptámeros ofrecen grandes ventajas, como su fácil producción *in vitro*, las modificaciones químicas que pueden ser incorporadas para mejorar su estabilidad en las muestras biológicas, el reconocimiento de blancos inmunógenos y no inmunógenos, su estabilidad a altas temperaturas y su capacidad de renaturalizarse. Todas estas características los convierten en una gran alternativa diagnóstica y terapéutica. En la última década, ha aumentado el interés por investigar estas moléculas, lo que se ve reflejado en el número de publicaciones científicas y patentes concedidas que contienen el término *aptamer,* además de los estudios clínicos que se llevan a cabo en estos momentos en todo el mundo.

Los blancos que pueden ser reconocidos por los aptámeros son tan variados y complejos que no hay límite para la cantidad de técnicas que podrían crearse en el campo de la parasitología. Larry Gold, uno de los creadores de los aptámeros, mencionó en una edición especial publicada por el *Journal of Molecular Evolution* en el 2015 que …“[…] el futuro del uso de los aptámeros se verá limitado solo por nuestra imaginación […]” ^77^*.*

Los aptámeros mencionados en esta publicación evidencian cómo estas novedosas moléculas pueden usarse como potenciales herramientas diagnósticas, terapéuticas y de investigación para el tratamiento de infestaciones parasitarias como la malaria, la leishmaniasis, la amebiasis, la tripanosomiasis y la criptosporidiosis.

En el caso del paludismo, los aptámeros obtenidos contra la PfLDH han derivado en la creación de un biosensor para el diagnóstico de la infestación por *P. falciparum.* Los demás aptámeros mencionados se encuentran en fase de prueba como herramientas diagnósticas o terapéuticas*.*

El tiempo necesario para obtener un aptámero y desarrollar un prototipo funcional, fluctúa entre uno y dos años. Por ello, se esperaría que en los próximos 10 años comiencen a aparecer comercialmente nuevas pruebas diagnósticas basadas en aptámeros. Los aptámeros que funcionan como alternativa terapéutica pueden tardar un poco más, ya que requieren ser probados en estudios con modelos animales y, posteriormente, en fases clínicas en humanos, pero el tiempo requerido es mucho menor en comparación con el cribado de compuestos químicos tradicionales.

El desarrollo pleno de esta nueva tecnología requiere de la atención de investigadores y de empresas públicas y privadas que contribuyan a la transición de las investigaciones a la etapa de desarrollo de prototipos comercializables y, ante todo, funcionales para las poblaciones vulnerables.

## Archivo suplementario 1

Lista completa de aptámeros en diferentes fases de desarrollo, que están siendo probados como herramienta terapéutica.

**Table:**

Title	phase
Pegaptanib Sodium in Patients With Wet Age-Related Macular Degeneration (AMD)	Phase 4
Anti-VEGF in Neovascular AMD	Phase 3
Fovista® (E10030) Intravitreous Administration in Combination With Lucentis® Compared to Lucentis® Monotherapy	Phase 3
Fovista® (E10030) Intravitreous Administration in Combination With Lucentis® Compared to Lucentis® Monotherapy	Phase 3
Fovista® (E10030) Intravitreous Administration in Combination With Either Avastin® or Eylea® Compared to Avastin® or Eylea® Monotherapy	Phase 3
Mg/Eye and 3 Mg/Eye Pegaptanib Sodium in Patients With Exudative Age-Related Macular Degeneration (AMD)	Phase 3
Pegaptanib Sodium, Compared to Sham, in Patients With Wet AMD.	Phase 3
Bevacizumab Versus Ranibizumab for Diabetic Retinopathy	Phase 3
Zimura® (Anti-C5 Aptamer) in Combination With Anti-VEGF Therapy in Subjects With Idiopathic Polypoidal Choroidal Vasculopathy (IPCV)	Phase 2
E10030 (Anti-PDGF Pegylated Aptamer) Plus Lucentis for Neovascular Age-Related Macular Degeneration	Phase 2
Phase II/III Study of Anti-VEGF in Neovascular AMD	Phase 2
An 18 Month Phase 2a Open Label, Randomized Study of Avastin®, Lucentis®, or Eylea® (Anti-VEGF Therapy)	Phase 2
Administered in Combination With Fovista® (Anti-PDGF BB Pegylated Aptamer)	Phase 2
Zimura in Subjects With Geographic Atrophy Secondary to Dry Age-Related Macular Degeneration	Phase 2
Study of the Safety, Tolerability and Pharmacokinetics of 1 Mg/Eye and 3 Mg/Eye Pegaptanib Sodium in Patients With Exudative Age-Related Macular Degeneration (AMD)	Phase 2
A Clinical Trial to Explore Safety and Efficacy of Different Doses of Pegaptanib Sodium, Compared to Sham, in Patients With Wet AMD.	Phase 2
Pegaptanib Sodium Compared to Sham Injection in Patients With DME Involving the Center of the Macula	Phase 2
A Study of the Pharmacokinetics, Pharmacodynamics, and Safety of ARC1779 Injection in Patients With Von Willebrand Disease Type 2B	Phase 2
ARC1779 Injection in Patients With Von Willebrand Factor-Related Platelet Function Disorders	Phase 2
IST Neoadjuvant Abraxane in Newly Diagnosed Breast Cancer	Phase 2
Lexaptepid Pegol (NOX-H94) in ESA-hyporesponsive Anemia in Dialysis Patients	Phase 2
A Study of AS1411 Combined With Cytarabine in the Treatment of Patients With Primary Refractory or Relapsed Acute Myeloid Leukemia	Phase 2
A Study of ARC1905 (Anti-C5 Aptamer) in Subjects With Dry Age-related Macular Degeneration	Phase 1
ARC1905 (ANTI-C5 APTAMER) Given Either In Combination Therapy With Lucentis® 0.5 mg/Eye In Subjects With Neovascular Age-Related Macular Degeneration	Phase 1
A Phase 1, Safety, Tolerability and Pharmacokinetic Profile of Intravitreous Injections of E10030 (Anti-PDGF Pegylated Aptamer) in Subjects With Neovascular Age-Related Macular Degeneration	Phase 1
Safety and Dosing Evaluation of REG1 Anticoagulation System	Phase 1
EYE001 to Treat Retinal Tumors in Patients With Von Hippel-Lindau Syndrome	Phase 1
An Open-Label Investigator Sponsored Trial to Investigate the Safety, Tolerability and Development of Subfoveal	Phase 1
Fibrosis By Intravitreal Administration of Altering Regimens of Fovista and Anti-VEGF Therapy in Subjects With Neovascular Age-Related Macular Degeneration	Phase 1
Lexaptepid Pegol (NOX-H94) in ESA-hyporesponsive Anemia in Dialysis Patients	Phase 1
NOX-E36 First-in-Human (FIH) Study	Phase 1
NOX-A12 First-in-human (FIH) Study	Phase 1
A Single-Center Trial of Intravitreous Injections of Macugen (Pegaptanib Sodium) Given at Least 7 Days Before Vitrectomy Secondary To Tractional Retinal Detachment in Proliferative Diabetic Retinopathy	Phase 1
First-in-Human and Proof-of-Mechanism Study of ARC19499 Administered to Hemophilia Patients	Phase 1
NOX-A12 Multiple Ascending Dose Study in Healthy Volunteers	Phase 1
Iloprost in Preventing Lung Cancer in Former Smokers	Phase 1
Alsertib (MLN8237) and Brentuximab Vedotin for Relapsed/Refractory CD30-Positive Lymphomas and Solid Malignancies	Phase 1
The Clinical Application of 68Ga Labeled ssDNA Aptamer Sgc8 in Healthy Volunteers and Colorectal Patients	Early Phase 1
